# Contraceptive preferences and use among auto artisanal workers in the informal sector of Kumasi, Ghana: a discrete choice experiment

**DOI:** 10.1186/s12978-015-0022-y

**Published:** 2015-04-12

**Authors:** Peter Agyei-Baffour, Mary Yaa Boahemaa, Ernestine A Addy

**Affiliations:** Department of Community Health, Kwame Nkrumah University of Science and Technology (KNUST), College of Health Sciences, School of Medical Sciences, Kumasi, Ashanti Ghana

**Keywords:** Contraceptive use and preference, Contraceptive attributes, Informal sector operators, Artisanal auto mechanic, Ghana

## Abstract

**Background:**

Contraceptive uptake in Ghana, especially in the Ashanti region remains low. This may be partly due to products’ characteristics and choice which are influenced by attribute utility trade-offs by consumers in determining which method offers the optimal combinations, given the needs and desires of the individuals making the choice. The study sought to determine how specific attributes of contraceptives influence artisanal auto mechanics’ stated preferences for a hypothetical contraceptive use in the Tafo-Suame industrial area of Kumasi, Ghana.

**Methods:**

A discrete choice experiment was conducted with artisanal auto mechanics in the study area from May to September 2011. Based on the summary of the attributes from the focus group discussion and in-depth interviews preceded administration of structured questionnaire, a discrete choice experiment (DCE) was created. The attributes used were; side effects, reversibility, ease of use, ability to prevent both pregnancy and sexually transmitted infection (STI’s), price and privacy in acquiring and attractiveness of the method. A total of 340 consented respondents aged 15 to 49 years participated in the study. Data were entered in Access and Sawtooth software SSI Web CAPi module and then exported to Stata for analysis.

**Results:**

The study showed a universal (99.4%) knowledge on contraception, ever used 87% and currently using a method, 58%. The study revealed that methods’ reversibility (β = 21.74; 95% CI: 20.17, 23.3), minimal allergic reaction (β = 13.93; 95% CI: 12.8, 15.05) and no effect on blood pressure (β = 12.71; 95% CI: 11.62, 13.79), were strongly associated with contraceptive preference and use. While contraceptives’ ability to prevent “only pregnancy”, (β = -15.13: 95% CI: -16.2, -14.02; “only STI’s”) (β = -11.65; 95% CI: -11.84, -11.46); and interrupt during sexual activity (β = -4.26; 95% CI: -5.19, -3.34), had large negative influence on contraceptive preference and use.

**Conclusion:**

The study has documented the magnitude of the effects of contraceptive attributes on informed choice, use, preference. It revealed that reversibility, side effects and ability to prevent both pregnancy and STI’s are the major important attributes that. The findings have implications on contraceptive development, uptake and the implementation of other family planning programmes.

## Introduction

### Contraceptive choice

Worldwide, each year, close to 350,000 women die and another 50 million suffer illness and disability from pregnancy and child birth complications [1]. There is evidence to suggest that effective contraceptive uptake prevents an estimated 2.7 million infant deaths and the loss of 60 million of healthy life in a year [2] while promotion of family planning could reduce poverty and hunger and avert 32% of all maternal deaths and nearly 10% of childhood deaths [3].

Contraceptive choice is integral in the provision of family planning services and plays an important role in women’s reproductive rights [4]. Informed choice and effective use of contraceptive methods could improve the health status, yet, in Ghana, coverage of family planning services is very low, especially in the Ashanti region with a coverage rate of 17.5% [5]. It is important to note that, choosing a contraceptive method involves assessing all the available methods in terms of its characteristics and determining which method offers the optimal combination of characteristics [6]. Making a choice presupposes having ample knowledge about suitable contraceptive methods which is also a pre-requisite of access and use. Generally, the principle of informed choice focuses on the individual yet, most people’s family planning decisions also reflect a range of outside influences [7-9].

Contraceptive as used in this study is defined as per the 2008 Ghana Demographic and Health Survey (GDHS) to include methods and practices that aim at delaying pregnancy and child birth, spacing children, limiting family size, preventing unintended pregnancies and eliminating the need for induced abortion [10]. Contraceptive use like any commodity is related to the various attributes possessed by the method under consideration in the circumstance in making a choice. The United States Agency for International Development (USAID) Population reports in 2005 revealed demand for more choices among users and providers of family planning. Consumers wanted contraceptive methods that could provide highly effective protection and at the same time causing fewer side effects, costing less, and are very easy to use. In response, manufacturers, researchers and providers of family planning are stepping up their efforts to improve existing contraceptives to offer a wide range of safe, effective, and convenient methods and service to encourage effective contraception.

Snow et al. [11], also indicated expression of substantial dissatisfaction in the available modern contraceptives among women from various nationalities but little or no evidence exist on the situation among male dominated population or occupational groups such as artisanal auto mechanics. Users and non-users of family planning services emphasized that the available methods often caused physical discomfort, were cumbersome to use and do not work effectively while the principal reasons and attribute that informed the choice of a method was its effectiveness and shorter reversible interval [11,12].

For attributes such as privacy in acquiring and using a method, there were regional contrasts in the extent to which women valued this citing that the contraception decisions ought to be jointly made.

A series of cross-sectional surveys conducted among samples of different occupational groups in Hong- Kong about condoms use as a strategy for preventing human immuno-deficieny virus (HIV) infection and sexually transmitted diseases (STDs) revealed similar interesting findings. The study showed that, of the 1508 respondents, 52% were married and aged lesss than 45 years. Overall, 24% of respondents reported they used condoms always and 22% reported that they never used condoms, thus the 76% of respondents were classified as non-users of condoms. Among all the respondents, the most common reason for condom use was to delay pregnancy (70%), prevention of STDs (46%) and AIDS (36%) and partner’s desire (10%) citing sexual obstruction and side effects as reasons for non-use [12]. Information on contraceptive use among occupational groups, especially auto artisanal mechanics, a popular industrial hub in most large cities in Ghana including Kumasi is scanty.

Studies in Tehran and Bombay revealed the most common reasons for halting the use of modern contraceptive methods as dissaproval by spouse and the fear of becoming pregnant during a stipulated time [13,14] while in Nepal a similar study showed a high usage of modern method at 125 (86.8%) among female partners and 12 (8.3%) following a natural method. But 65 (45.1%) were quick to state that male contraceptives interfered with sexual satisfaction, fear of side effects and the need to procreate as partners were disincentive to use [15].

Similar trends of unmet contraceptive needs have been documented among married men in urban areas in Nigeria with reasons for non-use of contraceptives as; fear of side effects (30%), religion (20%) and other attributes, doubts of effectiveness, uncomfortable side effects and reversibility [16,17] while in Ghana similar reasons have been found among the genearal population.

Although, Ghana was among the first sub-Saharan African countries to adopt a population policy, in 1969 to help in the achievement of a sizeable fertility rate, population is still growing, fertility, and STI’s including syphilis, gonorrhoea and AIDS are on the increase [18]. Post contraceptive campaign findings have revealed a huge knowledge-practice gaps in contraceptive use. While knowledge of contraceptive methods is almost universal, 98% of all women and 99% of all men in Ghana, the level of patronage of family planning services still remains low at 27% [19], acceptance rate falling from 33.8% (2008) to 31.1% in 2009 with Ashanti region having the lowest 17.5% [20]. It is undeniable fact that the provision of effective family planning services cannot be made on speculations and misconceptions. Yet there is limited evidence on what motivates clients’ use and preference for contraceptives, especially among occupational groups such as auto artisanal mechanics. One of the methods that has been used to assess consumers’ choice decisions of commodities is the discrete choice experiment [21].

### Application of discrete choice experiments (DCEs)

The DCE is increasingly being used in health economics, gaining widespread use in health care and has been applied successfully in several areas such as eliciting patients’ and the community’s preferences in the delivery of health services; establishing consultants’ preferences in priority setting; developing outcome measures; determining optimal treatments for patients; evaluating alternatives within randomised controlled trials; and establishing patients’ preferences in the doctor-patient relationship [21-23] and analyzing motivation of accepting rural postings among medical, nursing and midwifery students [24-26].

The technique is an attribute-based measure of benefit, based on the premise that, any good or service can be described by its characteristics (or attributes) and that the extent to which an individual values a good or service depends on the levels of these attributes. Within a DCE, respondents are asked to choose between two or more alternatives guided by their attributes. Resulting choices reveal an underlying or latent utility function. The approach combines random utility theory (RUT), consumer theory, experimental design theory and econometric analysis [27].

A crucial aspect of constructing a DCE is defining the choices. A full factorial design elicits preferences for all combinations of attributes and levels. This often results in a large number and experimental design methods used to create smaller fractional factorial designs (FFDs). When employing FFDs the researcher selects a set of choices (experimental design) which enable main effects (i.e. the effect of each independent variable on the dependent variable) and possible interactions (i.e. preferences for one attribute depend on the level of another) to be estimated.

Thus responses from choices are modelled within a RUT framework. As shown in Equation 1, the latent utility of an alternative *i* in a choice set *C*_*n*_ (as perceived by individual n) is decomposable into two additively separable parts:

I. a systematic (explainable) component specified as a function of the attributes of the alternatives V(Xin, ß); and
II. a random (unexplainable) component *ε*_*in*_ representing unmeasured variation in preferences.

The equation 1 is, ${U}_{in}=V\left({X}_{in},\beta \right)+{\varepsilon}_{in};$ It is assumed that individual *n* will choose alternative *i* if that alternative maximises her utility among all other alternatives in the choice set C_n_.

Ryan & Farrar’s study on using conjoint analysis to elicit preference for health care conducted in [28] stated that DCE is mainly used to assess willingness to trade between characteristics in optimal decision making in resource poor setting, produce overall benefit scores for alternative ways of providing health care; estimate the relative importance of different characteristics of a service for policy impact of each characteristic on overall benefit and estimate whether an attribute is important especially in assessing the outcomes of trials.

In applying these to contraceptive use, the study sought to assess how consumers could be motivated beyond mere increased knowledge on contraceptives to a change in attitude and increase in appropriate practice of contraceptive usage which is presumed to have the capacity to improve the current situation of low acceptance of contraceptives in Ghana. Following this, it was hypothesized that beyond universal knowledge, the makeup or attributes of contraceptives predict individual preference and use. Therefore it is important to know the sets of combination of contraceptive attributes that occupational groups, specifically, artisanal auto mechanics would prefer through a quantitative exploration of their effects on choice.

## Methods

### Study methods and design

A descriptive cross-sectional study which employed both qualitative and quantitative techniques was conducted from May to September 2011. The qualitative study preceded the quantitative study. First, focus group discussions and in-depth interviews were conducted with artisanal auto mechanics to elicit important attributes that informed choice of contraceptives. These attributes were very essential aids for the design of a Discrete Choice Experiment (DCE).

As explained earlier, DCE is a quantitative technique used in evaluating the relative importance of different product attributes that influence consumers’ choice behaviour [29]. In such experiments respondents are asked to state their choices over hypothetical or theoretical alternative settings, described by several attributes cited by them. These attributes are then put into various levels to show their relative strengths. It was adopted in this study to analyse the influence of certain stated attributes on the choice of a contraceptive method. It was assumed that the attributes were constant in each choice scenario but the levels that described each attribute could vary across scenarios. Analysis of the options chosen by respondents in each scenario revealed the extent of importance of each attribute to consumers’ preference and choice of a contraceptive method.

### Study area

The study was conducted in Kumasi in the Ashanti Region due to its cosmopolitan area having diverse tribes or ethnic groups in the Ghana and other nationals which make it appropriate for policy input. Although the indigenous Asante people dominate life in general, especially in commerce and industry, these migrant communities maintain their language and cultural identities. Asante Twi is universally spoken and understood. Kumasi is a bustling commercial, academic and recreational centre with a vibrant logging and lumber industry, general commerce and auto repairs which have distinct geographical locations such as Suame –Tafo magazine (the largest), Asafo Magazine and Sofoline Magazine. It also possesses major characteristics of Ghana, so findings from the study are likely to be extrapolated to represents what pertains in most Ghanaian communities. Kumasi is the second largest city in Ghana and it is the administrative capital of the Ashanti Region. It has an estimated population of 1,581,141 with an annual growth rate of 3.4%. Males constitute 49% and females 51% [30]. Politically, the metropolis is divided into ten (10) sub-metros. The study was conducted in the Tafo-Suame in the Manhyia North sub-metro in the Kumasi metropolis which houses the largest artisanal auto mechanic industrial area (Tafo-Suame) in Ghana [30].

The Tafo-Suame Industrial Area is an artisanal engineering cluster covering an area of over 50 km^2^ in the city of Kumasi. Its development dates back to 1929 and has constantly increased in size over the years. It has being one of the largest industrial areas in Africa. The Suame Magazine as is popularly called represents the largest concentration of auto-mechanics in West Africa. Over 200,000 people live and work in this area and approximately 12,000 independent micro, small and medium sized enterprises are located in the area. Tafo-Suame magazine is mostly gathered around the automotive sector, which involves car repairs and sale of spare parts and other activities complementary to auto mechanics. Most of the businesses are very small and specialized like most private informal sector entities.

The working population of Suame Magazine is considered informal sector operators. The sector covers a wide range of activities that meet local demands for a wide range of goods and services. The economy of Tafo-Suame magazine is dominated by sole proprietorship, cottage industries, apprentices and self-employed artisans. There are manufacturers of vehicle parts, traders, transporters, tailors, shoe makers, mechanics, electricians, plumbers and many others. The people tend to use less capital per worker than as in large firms. Medium and Small scale Industrial setting make use of local materials and recycle materials which would otherwise be considered as waste or scraps providing low-cost practical on-the job training. There are service industries; mean firms or enterprise whose final output is non – material irrespective of the type of occupation within the firm or enterprise located in the area. As the biggest industrial estate in Africa, Tafo-Suame Magazine has business activities that result in daily cash financial transactions of over US$1,000,000 on the lower variant of US$5 per person per day [31]. These characteristics are likely to attract sexual trade or negotiations of sexual activities and marrying of more wives as artisanal auto mechanics are relatively richer. Hence targeting them for attitudinal change for improve use beyond knowledge of contraceptive is important.

### Study population and sampling

Contraceptives strategies have over targeted female respondents at the expense of males. But in Ghana and most part of developing countries, decision to use contraceptives or not is strongly influenced by males’ choice. Hence this study used a male dominated study population or occupational groups such as artisanal auto mechanics. The respondents for this study were selected from auto mechanics in the Tafo-Suame Industrial area in their reproductive ages. Auto mechanics as stated in the study includes all artisans who work directly as auto mechanics or whose works complement the work of auto mechanics. The inclusion criteria in this study were being Ghanaian auto mechanic, or providing support service, consented to participate, aged 15-49 years as revealed by the focus group discussions and attendants of pharmacy or chemist shops. All respondents who did not meet these criteria were excluded. It is important to note that a majority of respondents were males and therefore the study did not necessarily consider gender as important independent variable for contraceptive use but rather tested whether large occupational groups such as artisanal auto mechanics could be targeted for improved use.

A sample of 340 artisanal auto mechanics were involved in the DCE. This figure was arrived at using the following assumptions and a formula, $\mathrm{n}=\underset{\bar{\mkern6mu}}{{\mathrm{Z}}^2\rho q/}\;{\mathrm{d}}^2$ by Kirkwood and Sterne [32] where n = the desired sample size z = the reliability coefficient of 1.96, that is 95% confidence interval, p = the proportion of artisan auto mechanics currently using any contraceptive method i.e., 27% or 0.27 [30], q = the allowable error margin, 1.0-p, i.e. (1.0-0.27) = 0.73, d = degree of accuracy desired at 0.05 and 10% of non-response. This resulted in 333.3 rounded up to 340 to account for sampling errors and variability.

#### Sampling of respondents

The study area was divided into 4 × 85 clusters using the main routes; Kumasi-Techiman, Kumasi-Mampong, Tafo-Maakro and Suamae-Bantama. Eighty-five artisanal auto mechanics were selected from each cluster. In the selected workshops, at least two people were interviewed on the basis of age or status, that is, those who had worked for more than a year or being a senior or junior apprentice. Respondents were made to select from a ballot box and those who picked ‘Yes’ were made to sign informed consent and enrolled into the study. This was repeated till the required sample size was arrived at. The study encouraged voluntary participation with the data gathered having representation from every section of the industrial area.

### Data collection

A pilot study using a sample of twenty (20) auto mechanics were selected from Sofoline magazine, another area that has artisanal auto mechanics in the Kumasi metropolis to assess the validity and reliability of the data collection tools. The pretest gave the opportunity to assess the reactions of respondents to the research procedures, such as the duration of interviews and reliability of data collection tools, ability of respondents to accurately understand questions asked as well as the ability of field assistants to ask and record the right questions and answers accurately. It further provided information on proportion of male to females in auto artisanal mechanics (which showed male dominance with females providing support services). Questions in both qualitative and discrete choice experiment were formed around the main themes of the study such as knowledge of contraceptives, sources, practice, contraceptive choices or options available, attributes, use and factors that influence choices, Other factors that were taken into consideration included the appropriateness and sequence in which questions were arranged, time allocated to the interviews and the budgetary implications of the study. All research assistants were involved in the pre-test exercise to prepare them adequately for the field work. Necessary corrections were made to the questionnaires before the actual data collection exercise was conducted.

To determine which hypothetical scenarios were suitable for the DCE, five focus group discussions (FGDs); four male and one female groups with each group consisting of 6-8 participants were conducted prior to the discrete choice in the study area to solicit for their views on the attributes they thought were important determinants of their choice of a contraceptive. A focus group discussion guide was used to interview participant to assess their views on and knowledge of contraceptive methods and priority areas they wanted a change in. The interview was conducted in the Asante Twi dialect.

Seven contraceptive attributes were shortlisted for potential inclusion in the DCE based on literature and their importance to the consumers (gained through the number of times they were mentioned during the focus group discussion). These attributes were; price, ease of use during sexual intercourse, ability to prevent pregnancy, STI’s/HIV, side effects, reversibility, attractiveness of the package and confidentiality. Levels were established for each attribute to reflect current conditions and additional levels to represent reasonable improvement in the base situation.

Eight (8) scenarios were selected using the fractional factorial design and technique. The questionnaire also included questions on respondent’s socio-demographic characteristics and their knowledge on and usage of contraceptives. The questionnaires were administered by trained field assistants through face- to-face interviews with auto mechanics in the various clusters of the area using pencil and paper and later entered into Sawtooth software SSI Web CAPi module and exported to Stata for data analysis.

### Data analysis

Qualitative data were thematically analysed. Recorded tapes were transcribed verbatim and translated into English. Transcripts were coded and summarized after authors have read them. Meeting was then held by authors to agree on themes for analysis. The attributes that were used for the design of the questionnaire and DCE were deduced from the qualitative data.

The main variables of interest were contraceptive preference and use as dependent while the covariates included age, religious affiliation, marital status, educational level, income level, cultural beliefs, prevention of STI/ HIV, perception about effectiveness and associated side effects, cost, reversibility and attractiveness.

Data collected using structured questionnaire were entered in a pre-designed template in Microsoft database, Access 2010. The entered data were cleaned by cross checking with the questionnaires to ensure accuracy and consistency in the responses and also minimise data entry errors. The DCE was programmed using SSI Web CAPI Module (Sawtooth Software Inc., Sequim, WA, USA). The data were then imported into STATA vs. 11 (Stata Corporation, Texas, USA) for analysis. Frequencies, percentages, means, and standard deviations were calculated for the demographic characteristics and the knowledge, attitude and use of contraceptives among respondents using Stata’s univariate descriptive statistics commands. Logistic regression was used to assess associations between background characteristics and other independent variables and the current use and non-use of contraceptives.

The DCE modelled the attributes and their effects on the contraceptive use using statistical procedures such as mixed logit models and multinomial and conditional logistic regression to indicate the strengths of the effect of an attribute on the probability of choice of contraceptive method made by respondents. Mixed logit models accounted for the demographic characteristics of respondents through the inclusion of interaction terms between the demographic variable and the stated attributes. Salient quotes from the qualitative surveys were used to further explain results from the discrete choice experiment and quantitative findings.

### Ethical considerations

The study protocols were reviewed and cleared by the Committee on Human Research, Publication and Ethics (CHRPE), an institutional review board of the Kwame Nkrumah University of Science and Technology and Komfo Anokye Teaching Hospital. Introductory letters were sent to both the Metropolitan Health Directorate (MHD) and Suame Magazine Industrial Development Organisation (SMIDO) during which the intent and procedures of the study were explained. Participants’ were made to sign or thumb print Informed Consent forms before the commencement of the interviews and FGDs. To ensure confidentiality of information supplied, the questionnaires were made anonymous by assigning only identification numbers and not names. Respondents had the freewill to enroll or not to enroll onto the study. The tape-recorded discussions were destroyed immediately after transcription and transcripts destroyed after the analysis.

## Results

### Socio- demographic characteristics

The socio-demographic variables considered important in this study were age distribution, educational level, religion and marital status and income, Table 1. From the Table a total of 340 respondents consisting of 271 (79.7%) males and 69 (20.3%) females and aged 15-49 years were involved in the study. The mean age of the study participants was 31.1 with a Standard Deviation of 7.89. Out of the total number of respondents interviewed, 94.7% had had a form of formal education with a majority of 45.6% completing basic education. One hundred and twenty-seven respondents (37.4%) were married and a majority, 90.3%, were Christians. The average daily expenditure of the respondents was GH¢13.28 (USD 8.00), while average daily revenue stood at GH¢25.29 (USD 15.23), with one hundred and nineteen (35.0%) earning more than the average income.

**Table 1:** **Socio - demographic characteristics of respondents**

**Variables**	**Frequency (N = 340)**	**Percentage (%)**
Gender*		
Male	271	79.7
Female	69	20.3
**Age**		
15 – 19	12	3.5
20 – 24	56	16.5
25 – 29	104	30.6
30 – 34	58	17.0
35 – 39	47	13.8
40 – 44	37	10.9
45+	26	7.7
***Mean age: 31.12, SD: 7.89***		
**Educational level**		
No Schooling	18	5.3
Primary	37	10.9
Middle/JHS	155	45.6
SHS/Technical	118	34.7
Tertiary	12	3.5
**Marital status**		
Single	163	47.9
Married	127	37.4
Cohabitating	37	10.9
Divorced/widowed	13	3.8
**Religion**		
Christians	307	90.3
Moslems	30	8.8
Traditionalist	3	0.9
**Average expenditure**		
Less GH¢ 15	242	71.2
More GH¢ 15	98	28.8
***Mean expenditure: GH¢13.28, Range: GH¢ (2-50)***		
**Average Income**		
Less GH¢ 25	221	65.0
More GH¢ 25	119	35.0
***Mean Income: GH¢25.29, Range: GH¢ (2-200)***		

*Auto artisanal mechanics are mostly males; females provide support services such as sale of water, food, microfinance, sale of lubricants and other spare parts. SHS –Senior High School; JHS-Junior High School.

#### Practice of contraception

Information gathered on this section did not look at consistent use of methods but adoption of a method at a point in time, Table 2. About 88% had ever used a contraceptive while 58% were currently using a method. Condom and withdrawal methods were common representing 64% and 37% respectively.

**Table 2:** **Practice of contraception**

**Variable**	**Frequency (N = 340)**	**Percentage (%)**
**Ever Used any Method**		
Yes	281	87.8
No	59	12.2
**Current use of Method**		
Yes	186	58.1
No	154	41.9
Currently used Method*		
Condoms	119	63.98
Pill	8	4.30
IUD	1	0.54
Injectables	2	1.08
Rhythm	34	18.28
Withdrawal	68	36.56
Source of service* ^**1**^		
Government facility	12	5.61
Private facility	7	3.27
Chemical/ Pharmacy	195	91.12

*Multiple response included.

^1^Questions relating to methods heard about and sources of contraceptive information and service allowed for multiple responses.

#### Influence of contraceptive attributes on use

Consumers trade-off many alternatives guided by the attributes of the good or service in a choice decision due to limited resources to satisfy all their needs at a go. In the case of choosing a contraceptive method, attributes such as price, the look, reversibility and effectiveness of alternative methods like condoms, injectables, IUD are keenly considered in the minds of consumers.

From Table 3, respondents who had ever used a contraceptive method stated that, the reason for their choice of a particular method was mainly influenced by its perceived effectiveness (62.99%), reversibility (58.71%) and the method having fewer side effects (62.63%). Attractiveness of the method was not enough reason in respondents’ choice decision, this was evident as only 2.85% considered this in their choice. This was emphasized by participants during the focus group discussions as:

*“[…] ooh, cost of contraceptive is not all that important to me. So far as Im able to enjoy myself […..] hahahaaa! if a method has low negative side effects, cheap and easy to access, Im prepared to go for it.* (FGD, male group, Suame Magazine)

**Table 3:** **Contraceptive attributes and use**

**Variables**	**Frequency**	**Percentage (%)**
**Reason for Choice**		
Less Expensive	101	35.94
Effective	177	62.99
Less Side Effects	176	62.63
Reversible	165	58.71
Its Attractive	8	2.85
Received it for Free	8	2.85
**Reason for not using a method**		
Inconvenience	23	38.98
Afraid of Side Effects	46	77.97
Spouse Opposition	12	20.34
Religious belief	25	42.37
Sexually Inactive	20	33.90
Others	3	5.08
Important attributes for using*		
Price	45	13.24
Effectiveness	266	78.24
Reversibility	194	57.06
Side effects	297	87.35
Attractiveness	8	2.35

*Others were personal dislike and not aware of the method.

Respondents who were not using or had discontinued the use of contraceptive method cited; fear of side effects (77.97%), interference with sexual satisfaction or inconvenience, 38.98% and spouse opposing as the top three reasons. Other reasons were given as sexual inactivity, religion not knowing any method and the price of the method being expensive. This was again observed in the qualitative study when a participant disclosed:

[….] Mmmm, I look for how easy it is to use, availability and its ability to prevent STIs and pregnancy. Price is not all that important to me. (FGD, male group, Suame Magazine).

Consumers’ perception of prices of a contraceptive methods was described by 296 representing 91.13% as moderate and less expensive.

#### Predictors of choice of contraceptives in discrete choice experiment

The aforementioned analysis presumed that consumers chose contraceptives and use them on sight, as the results could not elicit the trade-offs in their choices. In this section, the study conceptualised that clients do not just choose from commodities on sight. Rather they trade-off utilities and or attributes of such commodities for which they make the choice. A discrete choice experiment was performed to assess these trade-offs. This is based on the assumption that, any good or service can be described by its attributes which the consumer evaluates before choosing it. The utility parameter estimates for the choice of contraceptive based on its attributes are shown in Table 4.

**Table 4:** **Results for discrete choice experiment on contraceptive use**

**Attribute**	**Parameter**		**95% confidence interval**
	**Mean** ^**1**^	**Std. dev** ^**2**^	
Price^3^	1.86	1.36	1.65 to 2.06
Interruption in act ^4^	-4.26	6.19	-5.19 to -3.34
Prevents STI’s Only^5^	-11.65	1.29	-11.84 to -11.46
Prevents Pregnancy only	-15.13	7.14	-16.2 to -14.07
Serious allergic reaction^6^	3.29	1.01	3.14 to 3.44
Minimal allergic reaction	13.93	7.55	12.8 to 15.05
No effect on BP	12.71	7.29	11.62 to 13.79
Reversible^7^	21.74	10.49	20.17 to 23.3
Colour coded^8^	1.8	0.62	1.71 to 1.89
Colourless	-0.35	2.38	-0.70 to 0.01
Privacy in acquiring^9^	1.19	1.35	0.98 to 1.39

^1^This represents the mean utility of each attribute conditional on other attributes in a choice set.

^2^Reflects the degree of heterogeneity among respondents in the utility of a given attribute.

^3^Continuous variable.

^4^Compared with no interruption with sexual activity.

^5^Compared with prevents both STI’s and Pregnancy.

^6^Compared with effect on BP.

^7^Compared with Irreversible.

^8^Compared with handy.

^9^Compared with no privacy in acquiring.

In the main effects model, reversible, minimal allergic reaction and no-effect on blood pressure were major predictors of choice of a contraceptive method among the auto-mechanics in the Tafo-Suame area. These were evident by the large positive co-efficients of the attributes; (β = 21.74; 95% CI: 20.17, 23.3) for reversible, (β = 13.93; 95% CI: 12.8, 15.05) for minimal allergic reaction and (β = 12.71; 95% CI: 11.62, 13.79) for no effect on blood pressure. This was also documented during the qualitative study as expressed by respondents as follows:

*[…] I have little knowledge about the negative side effects. Usually, health staff tell you only the good sides of the contraceptives and less on bad side effects. […] I know of contraceptives being able to prevent pregnancy and HIV/AIDS. But I do not know anything about the bad effects on me. […] I will not buy any good or service which will give me troubles. […] but I believe that if heath staff tell us the bad side too, it will help us in using it so I think they should inform us about these too!* (FGD, Male group, Suame Magazine)

Contraceptive methods’ ability to prevent pregnancy only and sexually transmitted infections (STI’s) only had adverse effect on consumers’ choice of a method as indicated by large negative coefficients. Contraceptives’ ability to prevent only pregnancy pulled a coefficient of (β = -15.13: 95% CI: -16.2, -14.02), whereas its ability to prevent only STI’s pulled (β = -11.65; 95% CI: -11.84, -11.46). Interruption in sexual activity also had an adverse effect on contraceptive choice as it pulled a negative coefficient (β = -4.26; 95% CI: -5.19, -3.34). Thus the auto mechanics were interested in methods that could prevent pregnancy, STI’s/ HIV and do not interrupt with their sexual intercourse. Prices, privacy, colour coded and attractiveness or the packaging in acquiring were not major predictors of consumers’ though they generated positive coefficients, they were not major attributes in choice decision because the coefficients were small.

#### Influence of price on contraceptive use in the discrete choice experiment

In most circumstances price of a commodity affects choice and use, especially if it is a normal good. In Table 5, the effect of price on clients’ choice of contraceptives was explored.

**Table 5:** **Analysis of the effect of price on other attributes**

**Price**	**Coefficient**	**95% confidence interval**	**P-value**
Interruption in sex activity	-0.028	-0.062 to 0.006	0.105
Pregnancy only	0.102	0.038 to 0.167	0.002
Serious allergic reaction	0.003	-0.077 to 0.083	0.936
Minimal allergic reaction	-0.054	-0.175 to 0.066	0.374
No effect on BP	-0.177	-0.285 to -0.069	0.001
Reversible	-0.092	-0.175 to -0.009	0.031
Colour coding	0.269	0.171 to 0.367	0.000
Colourless	-0.026	0.126 to 0.074	0.608
Privacy in acquiring	-0.08	-0.137 to -0.023	0.007

From Table 5, pregnancy only, no-effect on blood pressure and colour coding were statistically significant to the price of the contraceptive methods with p-values of p = 0.002; p = 0.001 and P < 0.001 respectively. Attributes such as interruption in sexual activity, minimal allergic reaction, no- effect on blood pressure, reversible had adverse effects on the price of the methods as they pulled negative coefficients. However, the influence of price on the other attributes was weak as most of the confidence intervals for the coefficients were zero.

## Discussion

Utilisation of family planning methods has significant socio-economic contributions to resource-poor setting countries [1,2,4] such as Ghana, yet uptake is low, especially in the Ashanti region [5,20]. The discrete choice experiment (DCE) has been used to assess clients’ willingness to trade-off characteristics in optimal decision making in resource poor setting, produce overall benefit scores for alternative ways of providing health care; in this case family planning services for policy impact of each attribute in assessing contraceptive uptake [21-23].

The study hypothesized that consumers’ choice for contraception is a function of trade-offs of utilities which are often represented by the attributes of health care such as the contraceptives or motivations [24,25]. Hence understanding the influence of these attributes of contraceptives on consumers’ choice will help bridge the knowledge-practice gap in contraceptives. The study sought to examine how consumers are motivated beyond mere increased knowledge on contraceptives to a choice decision making process and use, influenced by the product’s attributes through a discrete choice experiment (DCE) [22].

### Socio-demographic characteristics and contraceptive Use

The study revealed that socio-demographic characteristics such as age, gender, marital status, mean income and number of children can predict contraceptive use, however, the effects are not huge. Knowledge of contraceptive was universal as almost all respondents knew at least one method, with a few having adequate knowledge. The male condom and the pill were the commonly known and used methods. These findings are consistent with earlier studies on contraceptives in Nepal, Tehran, Nigeria and Ghana, which reported high knowledge level on contraceptives and the high popularity of condom among consumers [16-18,30].

The study like others revealed that the decision to use one contraceptive method over another is influenced by personal choice, perceptions of efficacy, personal risk, access, age, cost, level of education, ethnicity, marital status, current number of children, sexual orientation, pattern of sexual activity and level of cooperation between partners [18,23]. The most important consideration for use or non-use of contraceptives relates to clients’ perception of their own risk of pregnancy, its relevance in not getting pregnant, risk of sexually transmitted diseases and past history of contraceptive use [11,15,23]. Partner issues; monogamy or polygamy, power within the relationship to make contraceptive decisions, desire for children, and more importantly approval of or concerns about the choice of method [19].

In Snow and others [11] qualitative study and Topsever and colleagues study [12], a majority of women interviewed expressed substantial dissatisfaction with the available methods of modern contraception. Both users and non-users stated that available methods too often caused physical discomfort, cumbersome to use, or did not work effectively. Lengthy discussion of side effects in all sites underscored indications that use of a contraceptive, even sustained use over many years, could not be regarded as a sign of satisfaction with the method. Not only are many clients using methods they find disagreeable, but for some, the motivation to avoid pregnancy is so powerful that they are tolerating substantial discomfort.

### Influence of contraceptive attributes on use

In the study, it was revealed that the artisanal auto mechanics in the Tafo-Suame Industrial area valued higher the contraceptive attributes that have less side effects, are reversible and prevent pregnancy and STI’s. This is consistent with the results from the focus group discussions with both the male and female groups; the DCE analysis and Snow and others qualitative study on contraceptive attributes [6,10,13].

In our study, respondents stated that they did not know much about the side effects of the contraceptives they purchase and use, corroborating Shailesh et al. [14] Pandey et al., [15] and Oyodekun [17], where they found similar sentiments among women presenting at tertiary hospitals in Bombay, Nigeria and Nepal. In the results of the study, 87.35% expressed that the side effect was a major important attribute they considered in making a choice.

With regard to reversibility of the methods, many respondents expressed that it was a very essential attribute they consider in making a choice; most preferred a method which could easily be undone. The DCE analysis revealed that reversibility is the very most important attribute the respondents consider in making a choice. This was evident by the large positive coefficient it pulled (21.74; 95% CI 20.17, 23.3) but in assessing the importance of attributes to consumers, reversibility was the third most important with 57.06% stating it. This is consistent with Snow et al. and Grady et al. [6,11] who found concerns of method reversibility as frequently cited attributes of contraceptive technology among women.

### Predictors of choice of contraceptives

The high negative utilities of the prevent “pregnancy only” (-15.13; 95% CI -16.2, -14.07) and “STI only” (-11.65; 95% CI: -11.84, -11.46) options suggest that contraceptives’ ability to prevent both pregnancy and STI’s are considered pre-requisites for artisanal auto mechanics’ choice. This is most likely because the main uses of contraceptives are to prevent pregnancy and STI’s. These findings are consistent with data earlier collected on what respondents’ knowledge on contraceptive, where 82.2% stated they knew it for preventing pregnancy and 85.8% knew them for preventing STI including HIV/AIDS, and also confirmed by Holmes, Levine & Weaver [9] where condoms were described as effective for prevention of of STIs.

Contraceptive attractiveness and privacy in acquiring were not important attributes in the choice decision of respondents. This was evident by their coefficients of colour coded (1.8; 95% CI: 1.71, 1.89), colourless (-0.35; 95% CI: -0.70, 0.01) and privacy in acquiring (1.19; 95% CI: 0.98, 1.39). This is in line with study by Fevisetan & Ainsworth [23] and earlier analysis which assessed the important attributes of contraceptives to consumers, where price was important to 13.24% and methods attractiveness to 2.35% of respondents.

### Influence of income and price on contraceptive use

Low income levels have been found to be associated with difficulty in accessing contraceptive services in many resource-poor settings. Patients with low income find it difficulty paying for transportation costs and service charges to access contraceptives [23]. However, in the study, participants revealed that prices of the contraceptives were not major attributes to determine the use. This could be due to the fact that a majority of respondents earned regular income and more than the national minimum wage rate and thus had enough to spend on contraceptive services.

The analysis of the discrete choice experiment confirmed that price was not a major predictor of use even though it had a positive coefficient (1.86; 95% CI: 1.65, 2.06). This was also in line with the results on the important attributes to consumers where only 13.24% of respondents stated price as important.

The study has demonstrated the applicability of DCE in assessing the stated preference of clients in contraceptive use. This is consistent with studies in which DCE was used to achieve similar objectives with respect to other variables of interest such as preference for healthcare [28], preference for a genenral practitioner appointment [29] and stated motivations in accepting rural postings among medical students [24], midwifery students [26] and motivation crowding in medical students [25]. From the discussion, it is glaring that the attributes of contraceptives are very important in the choice decision making and subsequently uptake of contraceptives. Therefore in order to bridge the knowledge-practice gap in contraceptive use, it is imperative to refocus much attention on the make of contraceptives while sustaining the high knowledge level to improve uptake.

The study had some limitations. First, there could be some effects of social desirability on respondents’ responses, that is, some respondents may have responded to satisfy interviewers without necessarily reporting what they actually do. To minimize the effects of this, mixed methods; qualitative and quantitative methods were used to cross-check responses to same questions from different respondents. Although the requirements and use of contraceptives among males and females may be different, the study assumed that decision to use contraceptives or not depends largely on males’ approval and therefore did not analyse results of males and females separately. Unlike other studies, the study did not consider gender as important independent variable and thus use male dominated occupational groups. Future studies may consider comparative analysis of males and females and also use DCE and other analytical approaches to further explore these issues. The study could not validate the stated monthly income earned by respondents due to lack of income records, consequently, this may have been under or over stated. Grouping contraceptive attributes into only seven categories may have resulted in over simplification which could increase the magnitude of effects compared with if more than seven categories had been used. However this was consistent with earlier studies that used DCE. These limitations notwithstanding, the study design apart from being consistent with similar DCE studies cited in this paper also followed rigorous scientific procedures. Hence the effects of the limitations should be minimal and the findings could be validated and relied upon.

## Conclusions

The novelties in the study are manifold. First, it has applied the principles of DCE to assess the choice decision making of contraceptives. Secondly, it has documented the magnitude of the effects of some contraceptive attributes on choice and the fact that consumers’ choice for contraception goes beyond ample knowledge. Contraceptive choice or use is a function of trade-offs of utilities which are often represented by the attributes of the contraceptives. Important attributes that predict use of contraceptive are reversibility, minimal allergic reaction and effect on blood pressure. The methods’ ability to prevent only pregnancy and only STI’s had an adverse effect on consumers’ choice of method. Contraceptives that have the ability to prevent pregnancy only, no-effect on blood pressure and colour coding were statistically significant to the price of the contraceptive method.

## Abbreviations

CI: Confidence interval
DCE: Discrete choice experiment
FFDs: Fractional factorial designs
FDG: Focus group discussion
GHS: Ghana Health Service
GSS: Ghana Statistical Service
HIV: Human immuno virus
IUD: Intra uterine device
KATH: Komfo Anokye Teaching Hospital
KNUST: Kwame Nkrumah University of Science and Technology
MHD: Metropolitan Health Directorate
RUT: Random utility theory
SMIDO: Suame Magazine Industrial Development Organization
SOPs: Standard operation procedures
SPSS: Statistical Package for the Social Sciences
STDs: Sexually transmitted diseases
STIs: Sexually transmitted infections
USAID: United States Agency for International Development
USA: United States of America
